# Causal analysis of obstructive sleep apneas and immune cell variation: A 2-sample Mendelian randomization study

**DOI:** 10.1097/MD.0000000000043478

**Published:** 2025-07-18

**Authors:** Ming Ye, Yuanyuan Wei, Xinghua Tan, Peijun Liu

**Affiliations:** a Department of Emergency Medicine, General Hospital of Central Theater Command, Wuhan, China; b Department of Otolaryngology, EZhou Central Hospital, EZhou, China; c Department of Respiratory and Critical Care Medicine, The Central Hospital of Enshi Tujia and Miao Autonomous Prefecture, Enshi, Hubei Province, China.

**Keywords:** genetic association, immune cells, Mendelian randomization, obstructive sleep apnea syndrome, single-nucleotide polymorphisms

## Abstract

The intricate relationship between the immune system and obstructive sleep apnea syndrome (OSAS) is an area of active research, with conflicting findings regarding immune inflammation and OSAS. In this study, we employed a 2-sample Mendelian randomization approach using publicly available genetic datasets (ebi-a-GCST90018916 and finn-b-G6) to investigate the causal link between 731 immune cell characteristics and susceptibility to OSAS. We conducted comprehensive sensitivity analyses to ensure the reliability and consistency of our results and to mitigate the impact of confounding factors. Our analysis revealed a significant genetic association between OSAS and 3 specific immune cell traits: activated and secreting regulatory autophagic T cells, and absolute plasma blast/plasma cell count. These associations were statistically significant (*P* < .01) with odds ratios < 1, providing valuable insights into the complex interplay between immune cell dynamics and OSAS. These findings underscore the need for further clinical investigations to explore potential therapeutic targets and improve our understanding of the pathophysiology of OSAS.

## 1. Introduction

Obstructive sleep apnea syndrome (OSAS) is a prevalent sleep disorder characterized by repeated episodes of partial or complete upper airway obstruction during sleep.^[1,2]^ In the middle-aged population, the prevalence of OSAS is as high as 50% in men and approximately 25% in women. OSAS is associated with up to a 4-fold increase in overall mortality.^[3]^ However, a lack of symptoms and awareness often leads to an underestimation of its prevalence.^[4]^ These episodes can lead to frequent arousals, fragmented sleep, and a significant reduction in blood oxygen saturation. OSAS can significantly affect daily life by causing excessive daytime sleepiness and impaired cognitive function, and it may also lead to serious health complications such as cardiovascular diseases. According to the Global Burden of Disease research, it is estimated that millions of individuals worldwide are affected by OSAS.^[5]^ Currently, the primary treatments for OSAS include lifestyle changes, continuous positive airway pressure therapy, and surgical interventions.^[6]^ Early diagnosis and consistent management of OSAS are crucial to prevent its progression and the onset of associated comorbidities, thereby improving overall health outcomes.^[7]^

The relationship between OSAS and immune cells has garnered significant interest in immunology and sleep medicine research.^[8]^ Individuals with OSAS often exhibit alterations in immune function, particularly affecting T cell subsets and inflammatory cytokine concentrations. The severity of oxygen desaturation in OSAS is closely correlated with these immune modifications.^[9]^ Chronic and intermittent hypoxia resulting from OSAS can lead to oxidative stress, potentially altering the Th1 to Th2 cell ratio.^[10]^ Additionally, sleep disruptions and apneic episodes in OSAS may increase the levels of pro-inflammatory cytokines, such as tumor necrosis factor TNF-α and interleukin IL-6.^[11]^ Immunoglobulin E-mediated inflammation and irritant-triggered inflammation are crucial in OSAS, inducing swelling in the upper respiratory tract and exacerbating OSAS symptoms.^[12]^ Elevated CD4 T cell frequency in patients with OSAS is attributed to an upregulation of the nuclear protein Ki67, suggesting a connection between this pathology, inflammation, and Th2 immune responses. Moreover, OSAS can potentially boost NK and CD4 T cell proliferation capacity while impairing the capacity of neutrophils to uptake bacteria and generate reactive oxygen species.^[13]^ These findings suggest a potential link between OSAS and dysregulation of the immune system.

Mendelian randomization (MR) is a powerful epidemiological tool essential for inferring causal relationships from observational data, based on Mendelian inheritance principles. MR provides a methodological framework that adds robustness to causality determination.^[14,15]^ MR leverages genetic variants as instrumental variables (IVs) to infer causal relationships, thereby reducing confounding and reverse causation. This makes it a powerful tool for clarifying whether immune alterations in OSAS are a cause or consequence of the disease. In the realm of OSAS research, prior observational studies have highlighted several correlations between immune cell traits and OSAS, suggesting a plausible connection. However, observational studies are often limited by confounding factors and reverse causality, making it difficult to establish a definitive causal link. This study applies a 2-sample MR framework to assess the causal relationship between specific immune cell traits and the risk or progression of OSAS.

## 2. Materials and methods

### 2.1. Study design

**Figure 1.**
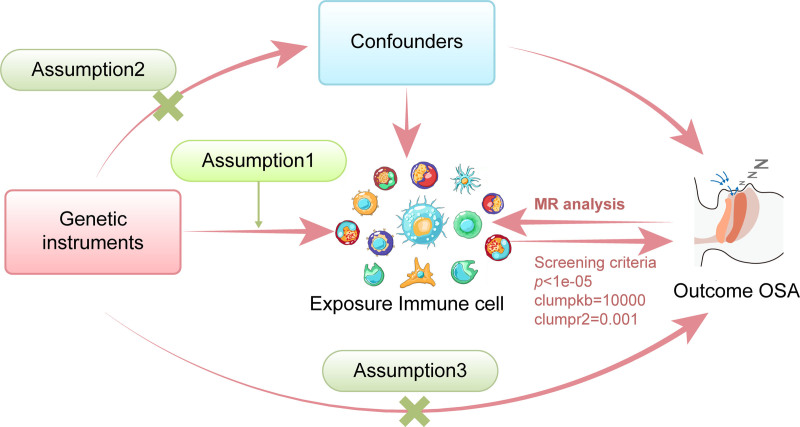
Illustration of the 2-sample MR analysis framework employed to investigate the causal relationship between 731 immune cell traits and OSAS. MR = Mendelian randomization, OSAS = obstructive sleep apnea syndrome.

We conducted a study to assess the causal relationship between 731 immune cell traits and OSAS using a 2-sample MR analysis (Fig. 1). MR leverages genetic variations as a proxy for risk factors. Thus, effective IVs in our causal inference had to satisfy 3 key assumptions regarding the genetic variants: they were directly associated with the exposure; they were not linked to potential confounders, and they influenced the outcome solely through the exposure, without being involved in any other pathways. To ensure these assumptions were not violated, we conducted a series of sensitivity analyses, including heterogeneity testing (Cochran *Q*), pleiotropy assessment (MR-Egger intercept and MR-PRESSO), and leave-one-out diagnostics. The studies included in our analysis were approved by the relevant institutional ethics review boards, and informed consent was obtained from all the participants.

### 2.2. Sources of genome-wide association studies (GWAS) data for OSAS research

GWAS summary statistics for OSAS were obtained from the ebi-a-GCST90018916 dataset. OSAS diagnoses in both datasets were primarily defined using ICD-10 code G47.3, supplemented by clinical features such as apnea-hypopnea index ≥ 5 when available.^[16]^ This study focused on a European population and included a total sample size of 476,853 individuals, comprising 13,818 cases and 463,035 controls. After quality control and imputation, approximately 24,183,940 single-nucleotide polymorphisms were analyzed in this study using the HG19/GRCh37 reference genome.

The validation dataset from the FinnGen database encompasses 38,998 OSAS cases and 336,659 control cases, primarily of European descent, providing a focused demographic for the study.

### 2.3. Genetic insights into immune cell traits

In this study, we included data from the GWAS catalog, analyzing approximately 22 million genetic variants affecting 731 immune cell traits in a cohort of 3757 Sardinian residents.^[17]^ This study identified 122 significant independent association signals for 459 cell traits across 70 genetic regions, including 53 novel discoveries. These results revealed various molecules and mechanisms involved in cell regulation. Additionally, 53 signals across 36 genetic regions overlapped with previously reported disease-associated signals, predominantly in autoimmune disorders, highlighting intermediate phenotypes in the disease process. These findings demonstrated the complex genetic regulation of immune cells and their specific impact on the risk of autoimmune diseases.

### 2.4. Selection criteria for IVs related to immune traits and OSAS

In genetic epidemiology, IVs were selected based on prior studies.^[18,19]^ The significance level for the immune traits was set at 1 × 10^−5^. SNP selection, using PLINK software, aimed to minimize linkage disequilibrium (LD *r*^2^ threshold < 0.001 within a 10,000 base-pair range), with LD *r*^2^ calculated based on the 1000 Genomes Project as a reference. For OSAS, the significance level was adjusted to 1 × 10^−5^. IVs with an F-statistic < 10 were excluded to ensure strength and reduce bias from weak instruments.

### 2.5. Statistical analysis

In this study, advanced statistical techniques in R version 4.2.3 were used to investigate the causal relationships between 731 immune phenotypes and OSAS. Inverse variance weighting (IVW) was prioritized as the primary method due to its efficiency and statistical power when IVs meet core assumptions. Weighted median, MR-Egger, and MR-PRESSO were employed as sensitivity analyses to assess the robustness of results and detect potential pleiotropy or outliers. The “TwoSampleMR” package was utilized for IVW and weighted median methods, revealing valuable insights into the genetic basis of the disease. Cochran *Q* test and *P* values were employed to assess variability across IVs. Additionally, methods such as MR-Egger and MR-PRESSO were applied to address the horizontal pleiotropy and outliers. Scatter and funnel plots were used to visualize the results and enhance their interpretation. Leave-one-out diagnostics reinforce the robustness of the findings, ensuring their reliability, even in the presence of potential anomalies. To balance discovery and control of false positives in this hypothesis-generating study, an false discovery rate (FDR) threshold of 0.20 was applied, with the understanding that the associations identified warrant further replication. Overall, these analytical approaches revealed significant associations between immune phenotypes and schizophrenia, validating our study results.

## 3. Results

### 3.1. Protective effects of immune phenotypes on OSAS

**Figure 2.**
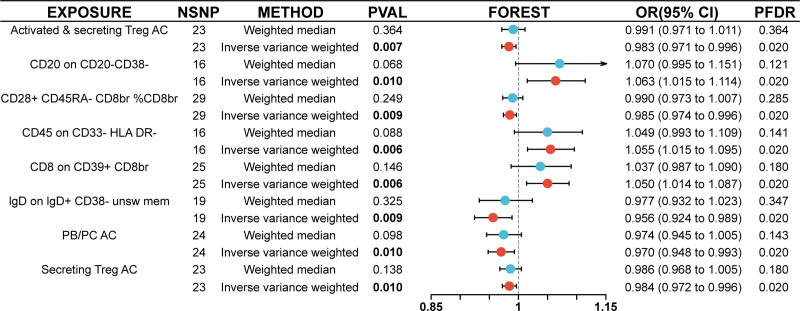
Graphical depiction of immune phenotypes protective against OSAS in the EBI dataset. EBI = European Bioinformatics Institute, OSAS = obstructive sleep apnea syndrome.

**Figure 3.**
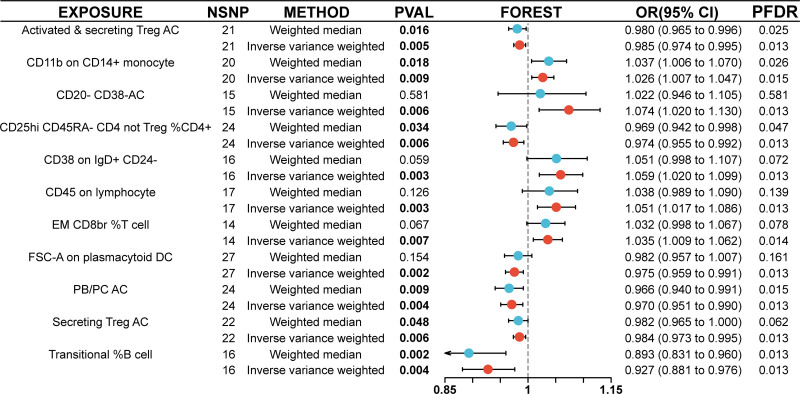
Graphical depiction of eleven immune phenotypes linked to OSAS in the FinnGen database. OSAS = obstructive sleep apnea syndrome.

In our study, prioritizing statistical accuracy through FDR adjustment (PFDR < 0.05), we identified immune phenotypes that exhibited protective effects against OSAS within the European Bioinformatics Institute dataset. The protective traits included but were not limited to activated and secreting regulatory autophagic T cells (Treg AC), CD20 on CD20−, CD38−, CD28+, CD45RA−, CD8BR, %CD8BR, CD45 on CD33− HLA DR−, CD8 on CD39 + CD8BR, IgD on IgD + CD38 − unsw mem, plasma blast/plasma cell absolute count (PB/PC AC), which reflects the quantity of antibody-secreting plasma cells, and secreting Treg AC, as illustrated in Figure 2. Further deepening our research, we focused on the FinnGen database, where we discovered an additional spectrum of 11 immune phenotypes associated with OSAS. This array included activated and secreting Treg AC, CD11b on CD14 + monocyte, CD20 − CD38 − AC, CD25hi CD45RA− CD4 not Treg %CD4+, CD38 on IgD + CD24−, CD45 on lymphocyte, EM CD8BR %T cell, FSC-A on plasmacytoid DC, PB/PC AC, secreting Treg AC, and transitional %B cell. The identification of these diverse phenotypes in the FinnGen database significantly enriches our understanding of the immune system’s role in OSAS, as depicted in Figure 3. This dual-database comprehensive exploration broadens our understanding of the immune mechanisms involved in OSAS, paving the way for future research.

**Figure 4.**
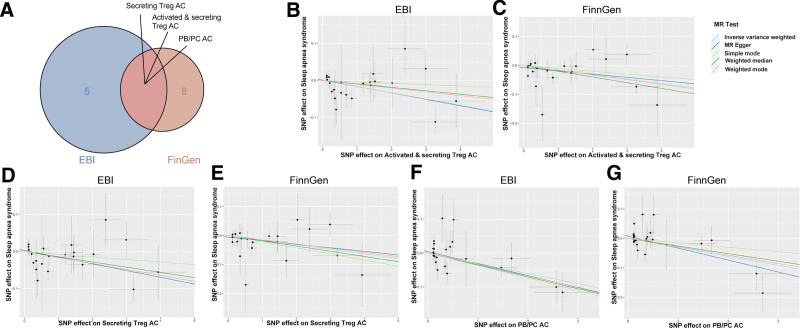
Visualization of IVW analysis results and scatter plot validations for the immune phenotypes showing protective effects against OSAS based on the EBI and FinnGen databases. (A) The protective roles of activated and secreting Treg AC, PB/PC AC, and secreting Treg AC, with odd ratios derived from the EBI and FinnGen datasets. (B–G) Scatter plots demonstrating the strong associations between immune cell phenotypes and OSAS were created to emphasize the robust nature of these relationships. AC = autophagy cells, EBI = European Bioinformatics Institute, IVW = inverse-variance weighted, OSAS = obstructive sleep apnea syndrome, PB/PC AC = plasma blast/plasma cell absolute count, Treg AC = regulatory autophagic T cells.

Cross-validation with the FinnGen OSAS data reinforced the protective roles of activated and secreting Treg AC, PB/PC AC, and secreting Treg AC. Using the IVW method, we obtained compelling evidence of their protective impact (Fig. 4A). In the EBI dataset, activated and secreting Treg AC presented an odds ratio (OR) of 0.983 (95% confidence interval [CI]: 0.971–0.996, *P* = .007), PB/PC AC had an OR of 0.974 (95% CI: 0.948–0.993, *P* = .010), and secreting Treg AC presented an OR of 0.984 (95% CI: 0.972–0.996, *P* = .010). Similarly, the FinnGen dataset revealed activated and secreting Treg AC with an OR of 0.985 (95% CI: 0.974–0.995, *P* = .005), PB/PC AC at an OR of 0.970 (95% CI: 0.951–0.990, *P* = .004), and secreting Treg AC at an OR of 0.984 (95% CI: 0.973–0.995, *P* = .006).

Our findings strongly indicate the fundamental importance of specific immune cell types in reducing the risk of OSAS. This assertion is supported not only by robust associations but also by rigorous validation, ensuring the absence of horizontal pleiotropy within these associations. This validation was meticulously confirmed by employing both the intercept of MR-Egger and global test of MR-PRESSO, ensuring the robustness and reliability of our results. To reinforce our conclusions on the 3 confirmed immune cell phenotypes, we employed advanced analytical techniques utilizing both scatter plots (Fig. 4B–G) and funnel charts (Figure S1, Supplemental Digital Content, https://links.lww.com/MD/P490). Although the effect sizes of individual immune phenotypes are modest, this is expected for complex traits such as OSAS. The cumulative and polygenic nature of immune regulation suggests that even small associations may collectively contribute to disease susceptibility.

### 3.2. Investigation into the causal impact of OSAS initiation on immunophenotypes

In our investigation, we employed a 2-sample MR analysis using the IVW method as the main statistical approach to examine the causal relationship between OSAS and immune phenotypes. After applying FDR adjustments, none of the immune traits reached the threshold of significance set at FDR < 0.05. However, by exploring a more liberal significance level of FDR < 0.20, we identified twenty immunophenotypes that showed potential associations (Figure S2, Supplemental Digital Content, https://links.lww.com/MD/P491). These immunophenotypes included the percentage of IgD + CD38dim B cells within the total B cell population; the percentage of CD11c + monocytes within monocytes; the absolute count of hematopoietic stem cells; the percentage of CD45RA− CD4 + T cells within CD4 + T cells; the percentage of naive CD4−CD8− T cells within CD4−CD8− T cells and within total T cells; the percentage of CD8dim T cells within total T cells; the absolute count and percentage of granulocytes within leukocytes; the percentage of CD28 + CD4−CD8− T cells, CD28 + CD45RA− CD8dim T cells, and CD28 + CD45RA− CD8 + T cells within T cells; the expression of CD86 on CD62L + myeloid dendritic cells; the expression of CD33 and HLA DR on CD14dim, CD14−, and the density of HLA DR expression on plasmacytoid dendritic cells, dendritic Cells, and CD33dim HLA DR + cells with CD11b + and CD11b− expressions. Although our bidirectional MR analysis did not reveal evidence of reverse causality, we acknowledge that OSAS-induced inflammation may still exert subtle or transient feedback effects on immune phenotypes, which warrant further exploration in future studies.

## 4. Discussion

Our research team employed publicly available genetic data to investigate the causal connections between 731 immune cell traits and OSAS. This groundbreaking study is the first to employ MR analysis to unravel intricate causal relationships between numerous immunophenotypes and OSAS. This revealed 3 specific immune cell phenotypes with a significant causal impact on OSAS, with an FDR <0.05, providing valuable insights into the underlying immunological mechanisms of this pathology and opening up new possibilities for potential therapeutic interventions.

Our research indicates that the number of activated and secreting Treg AC is positively correlated with the risk of OSAS. Tregs are specialized T cells that suppress the activity of other immune cells, preventing excessive immune responses and autoimmune diseases.^[20]^ When activated, Treg cells secrete immunosuppressive molecules, enhancing their ability to regulate immune responses.^[21]^ Activated and secreting Treg AC play a critical role in various physiological and pathological conditions, including inflammation, infection, autoimmune diseases, and cancer.^[20,22,23]^ In the tumor microenvironment, Treg cells help tumors evade immune surveillance by suppressing the immune system’s attack on tumor cells. In the context of OSAS, chronic intermittent hypoxia and systemic inflammation may drive the activation and expansion of Treg cells as a compensatory mechanism to counterbalance ongoing inflammation.^[24]^

PB/PC AC is used in immunology to quantify specific cell types derived from B cells.^[25]^ These cells play a crucial role in antibody production, essential for immune response. Plasma blasts are early-stage cells that differentiate from B cells after exposure to antigens. Monitoring PB/PC AC is essential for assessing the quantity of these key immune cells and the ability of the immune system to generate an appropriate immune response, offering valuable insights into individual immune status and response to various diseases, including infections, autoimmune disorders, and certain types of cancer. The relationship between plasma cells and OSAS is not yet fully clear.^[26,27]^ The relationship between Plasma Cells and OSAS is not yet fully clear. Plasma Cells are an integral part of the immune system, primarily responsible for producing antibodies to combat pathogens. OSAS is a respiratory-related sleep disorder characterized by repeated episodes of paused breathing and shallow sleep during the night.^[28,29]^ Hypoxia-induced inflammation in OSAS leads to the activation of hypoxia-inducible factor 1 alpha, which modulates immune responses by promoting pro-inflammatory cytokine production, such as tumor necrosis factor alpha, and altering T cell differentiation. This dysregulated immune environment can enhance regulatory T cell suppression, impair antigen-presenting cell function, and contribute to chronic systemic inflammation.^[30]^ Current research primarily focuses on the impact of OSAS on the immune system, especially its effect on inflammatory responses and immune regulation. Owing to the critical role of plasma cells in immune responses, OSAS may indirectly affect the activity of these cells by influencing inflammation and immune functions.^[31]^ Several OSAS-specific immunological studies have reported altered B cell and plasma cell profiles, supporting the notion that hypoxia-related inflammation may influence plasma cell dynamics in OSAS patients.^[32]^

Secreting Treg AC represent a unique subset of T cells responsible for maintaining immune tolerance and balance. Their main role is to prevent excessive immune responses and autoimmune diseases.^[33]^ Secretory Tregs secrete important immunosuppressive molecules, including IL-35 and IL-2, which play crucial roles in suppressing immune reactions and inflammation.^[34,35]^ Currently, the relationship between the active state of secreting Treg AC and OSAS remains unclear and requires further investigation. OSAS, which induces repetitive hypoxia and sleep interruptions, contributes to the development of the inflammatory response and indirectly affects immune cells, including Tregs. Therefore, OSAS might influence the overall immune response by altering the functionality or secretory characteristics of these cells.^[32,36,37]^ These findings suggest that targeting immune dysregulation, particularly the altered functionality of secreting regulatory T cells, could be a potential strategy for immunotherapy in OSAS, aiming to restore immune balance and reduce chronic inflammation. The observed increase in Treg and plasma cell levels in OSAS may reflect a compensatory response to chronic inflammation rather than a directly pathogenic role, highlighting the context-dependent nature of immune cell function in chronic diseases.

This MR study has some limitations. Genetic variants used as IVs may not capture all mechanisms linking immune cell traits to OSAS, and measurement errors in GWAS-based phenotyping could affect results. The complex nature of immune cell definitions and lack of data on cell functionality, such as cytokine production or activation state, limit interpretation. Our focus on European populations may reduce generalizability to other groups. Despite sensitivity analyses, residual confounding may remain. Further research with diverse populations and functional data is needed to confirm these findings.

## 5. Conclusion

Our bidirectional MR study revealed a causal relationship between specific immune cell phenotypes (activated and secreting Treg AC, PB/PC AC, secreting Treg AC) and the incidence of OSAS. Using IVs from extensive genetic data, we found that elevated levels of these immune cells were associated with an increased risk of OSAS. Our analysis showed higher ORs for OSAS development in individuals with increased levels of these immune cells. However, we did not find evidence supporting reverse causality from OSAS to immune traits, suggesting a likely unidirectional relationship. However, further studies are needed to confirm this finding.

## Author contributions

**Conceptualization:** Ming Ye.

**Funding acquisition:** Peijun Liu.

**Project administration:** Peijun Liu.

**Resources:** Xinghua Tan.

**Software:** Xinghua Tan.

**Visualization:** Yuanyuan Wei.

**Writing – original draft:** Ming Ye, Yuanyuan Wei, Xinghua Tan.

**Writing – review & editing:** Peijun Liu.

## Supplementary Material


