# Supplementary figures and images for: Causal analysis of obstructive sleep apneas and immune cell variation: A 2-sample Mendelian randomization study
Source: Medicine (Baltimore). 2025 Jul 18;104(29):e43478. doi: 10.1097/MD.0000000000043478 (PMC12282687; doi:10.1097/MD.0000000000043478)

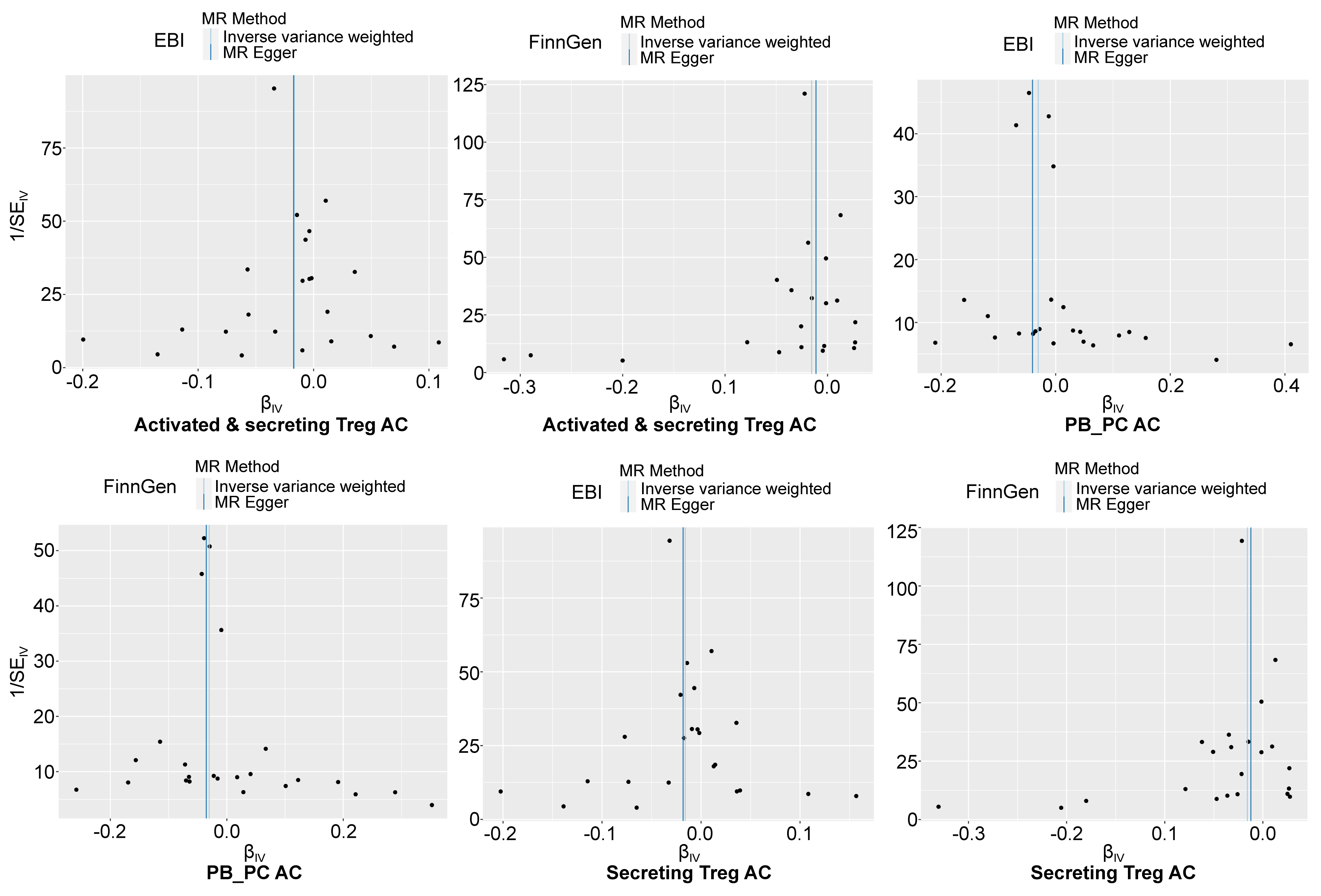

Supplement: Supplementary file 1 [file medi-104-e43478-s001.tif]

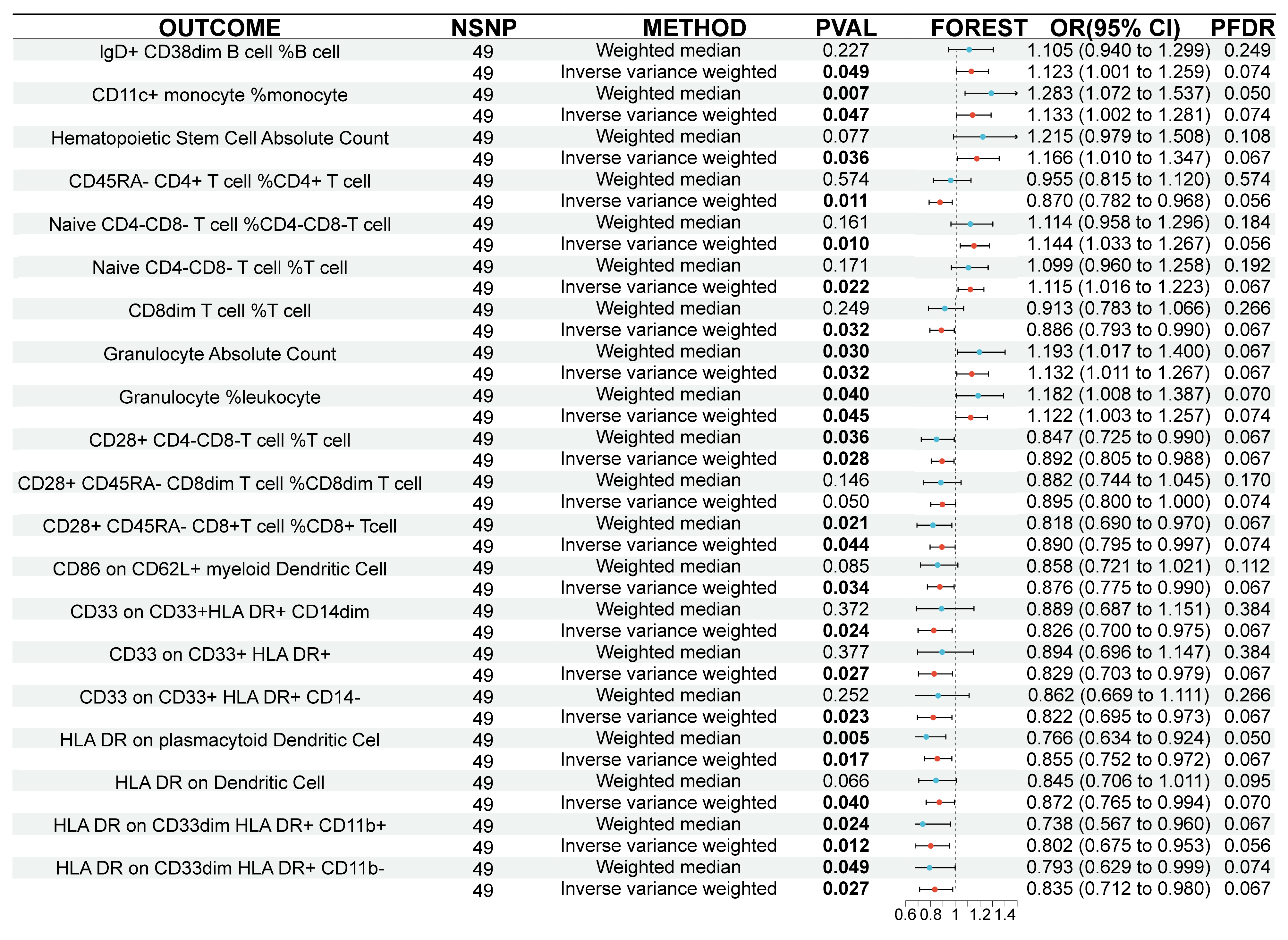

Supplement: Supplementary file 2 [file medi-104-e43478-s002.tif]
